# An Unexpected Mode Of Binding Defines BMS948 as A Full Retinoic Acid Receptor β (RARβ, NR1B2) Selective Agonist

**DOI:** 10.1371/journal.pone.0123195

**Published:** 2015-05-01

**Authors:** Eswarkumar Nadendla, Catherine Teyssier, Vanessa Delfosse, Valérie Vivat, Gunasekaran Krishnasamy, Hinrich Gronemeyer, William Bourguet, Pierre Germain

**Affiliations:** 1 Inserm U1054, Centre de Biochimie Structurale, Montpellier, France; 2 CNRS UMR5048, Universités Montpellier 1 & 2, Montpellier, France; 3 CAS in Crystallography and Biophysics, University of Madras, Chennai, India; 4 Novalix, Illkirch, C.U. de Strasbourg, France; 5 IGBMC, CNRS/INSERM/UdS/CERBM, Illkirch-Cedex, C.U. de Strasbourg, France; Nihon University School of Medicine, JAPAN

## Abstract

Retinoic acid is an important regulator of cell differentiation which plays major roles in embryonic development and tissue remodeling. The biological action of retinoic acid is mediated by three nuclear receptors denoted RARα, β and γ. Multiple studies support that RARβ possesses functional characteristics of a tumor suppressor and indeed, its expression is frequently lost in neoplastic tissues. However, it has been recently reported that RARβ could also play a role in mammary gland tumorigenesis, thus demonstrating the important but yet incompletely understood function of this receptor in cancer development. As a consequence, there is a great need for RARβ-selective agonists and antagonists as tools to facilitate the pharmacological analysis of this protein *in vitro* and *in vivo* as well as for potential therapeutic interventions. Here we provide experimental evidences that the novel synthetic retinoid BMS948 is an RARβ-selective ligand exhibiting a full transcriptional agonistic activity and activating RARβ as efficiently as the reference agonist TTNPB. In addition, we solved the crystal structures of the RARβ ligand-binding domain in complex with BMS948 and two related compounds, BMS641 and BMS411. These structures provided a rationale to explain how a single retinoid can be at the same time an RARα antagonist and an RARβ full agonist, and revealed the structural basis of partial agonism. Finally, in addition to revealing that a flip by 180° of the amide linker, that usually confers RARα selectivity, accounts for the RARβ selectivity of BMS948, the structural analysis uncovers guidelines for the rational design of RARβ-selective antagonists.

## Introduction

Retinoic acids and their analogs, referred to as retinoids, exert their pleiotropic effects through three retinoic acid receptor subtypes [RARα (NR1B1), RARβ (NR1B2) and RARγ (NR1B3)] that originate from three distinct genes [1–3]. RARs are members of the nuclear receptor (NR) superfamily [4] and act as ligand-inducible transcription factors binding to DNA regulatory elements in the promoter regions of target genes by forming heterodimers with another NR, the retinoid X receptor (RXR) [5, 6]. RARs are modular proteins composed of several domains, most notably the DNA-binding domain (DBD) and the ligand-binding domain (LBD). The well-established function of RARs is to regulate gene expression [5], and switching on RAR transcriptional activity relies on ligand-induced conformational changes provoking a dynamic series of coregulator exchanges. In the absence of agonists, RAR exerts a repressor activity by interacting with transcriptional corepressors (CoRs) which themselves serve as docking platforms for the recruitment of histone deacetylases that impose a higher order structure on chromatin that is not permissive to gene transcription. Upon agonist (i.e. the natural ligand all-*trans*-retinoic acid, atRA, or synthetic compounds that mimic its action) binding, conformational changes of the RAR LBD induce CoR release and favour the recruitment of coactivators (CoAs) such as CBP/p300, the p160 protein family, or CARM1 with histone acetylase activities allowing chromatin de-compaction and gene transcription [5, 6].

**Fig 1:**
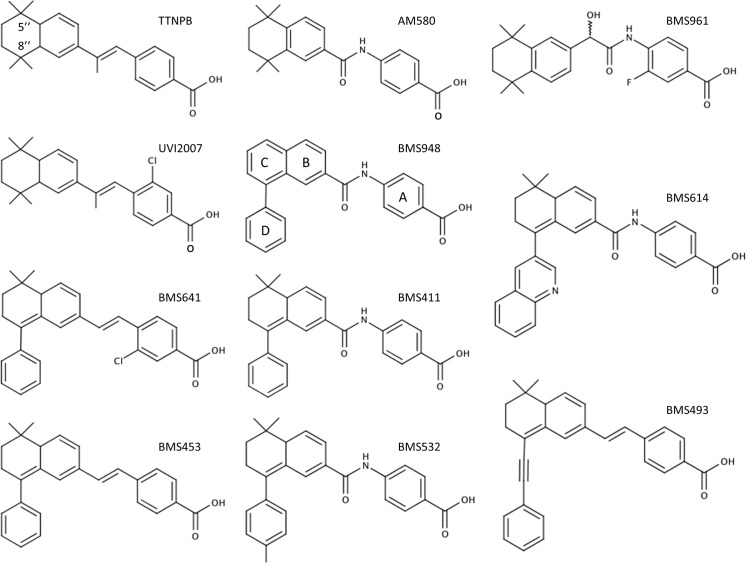
Chemical structures of the synthetic RAR ligands used in this study.

Our understanding of how ligand binding leads to receptor regulation has been greatly advanced by structural studies of RAR LBDs in complex with different pharmacological classes of retinoids as well as CoA- and CoR-derived fragments [7–12]. These structures revealed that RAR LBDs exhibit a common fold comprising 12 α-helices (H1-H12) and a short β-turn (s1-s2) arranged in three layers to form an antiparallel “α-helical sandwich”. This particular arrangement generates a ligand binding pocket (LBP) in the lower part of the domain. Another key component of the LBD is the C-terminal helix (H12) which is repositioned upon agonist binding to complete a hydrophobic surface with residues from helices H3 and H4 that is specifically recognized by the LxxLL motifs (also called NR boxes) of CoAs. Hence the interactions between H12 or residues in its vicinity and the bound ligands are critical for the control of the transcriptional activity of RARs. In this regard, the structures of RARα in complex with Am580 [12], BMS614 [8] and BMS493 [12] have revealed the structural basis of agonist, antagonist and inverse agonist action, respectively. Briefly, while Am580 (Fig 1) stabilizes H12 in the so-called active conformation described above, thus inducing CoA recruitment, the bulky extended 8”-quinolinyl group of the antagonist BMS614 (Fig 1) prevents CoA binding by redirecting H12 into the CoA binding groove. On the other hand, the inverse agonist BMS493 (Fig 1) which also contains a bulky extension prevents CoA recruitment and, in addition, reinforces CoR interaction by stabilizing a β-sheet interaction between the RAR LBD and specific CoR residues [12]. A fourth class of retinoids, referred to as partial agonists, consists in a group of molecules that fail to stabilize a particular conformation so their activity depends on the relative concentrations of cellular CoAs and CoRs. In the past twenty years, a large panel of retinoids with activities ranging from full agonists (any retinoid activating RAR as efficiently as atRA) to antagonists through partial agonists has been generated for potential therapeutic applications [13].

Despite their chemotherapeutical potential [14–18], the pharmacological use of retinoic acids is severely restricted because of their pleiotropic activities. The development of synthetic retinoids that specifically modulate only one RAR subtype might overcome these restrictions [17–19]. Note that a given ligand (agonist and antagonist) may be considered as selective when it exhibits an affinity difference greater than 100-fold between its primary target and other receptors (see International Committee of Pharmacology Committee on Receptor Nomenclature and Classification [4]). Under 100-fold difference the ligand may be defined as a compound that shows a preference for a given receptor. Comparison of RAR subtype sequences together with their 3D structures revealed only three divergent residues into their LBPs located in H3 (RARα Ser_232_, RARβ Ala_225_, RARγ Ala_234_), H5 (RARα Ile_270_, RARβ Ile_263_, RARγ Met_272_) and H11 (RARα Val_395_, RARβ Val_388_, RARγ Ala_397_) [20]. Ligands that display selectively or marked preferences for RARα or RARγ have been developed on the basis of specific hydrogen bonds formed with the polar Ser_232_ (H3) [21] and the weakly polar Met_272_ (H5) [9] in RARα and RARγ, respectively. However, no such discriminatory bond can be established in RARβ LBP so the development of RARβ-selective ligands is more challenging [10] and requires alternative strategies. There is a great need for RARβ-selective agonists and antagonists as tools to facilitate the pharmacological analysis of this protein *in vitro* and *in vivo* as well as for potential therapeutic interventions. In humans, there are three major isoforms for RARβ (β1 initiated at the P1 promoter, β2 and β4 initiated at the P2 promoter) harboring identical LBPs. The term RARβ in the literature usually refers to the RARβ2 isoform. Importantly, many studies have previously shown that RARβ2 possesses many of the functional characteristics of a tumor suppressor [22]. In some cellular models RARβ2 is required for the anti-proliferative effect of retinoic acid [23] and its expression is frequently lost in many neoplastic tissues [24–28]. Nevertheless, it has been recently reported that RARβ2 could also play a role in mammary gland tumorigenesis [29], thus demonstrating the important but yet incompletely understood function of this nuclear receptor in cancer development. At the present time, it has been possible to generate molecules with complex activities such as ligands that are RARβ agonists and RARα/RARγ antagonists [10, 20, 30, 31] or which exhibit only a partial agonistic activity toward RARβ such as BMS641 [10]. Here we report on the structural and functional characterization of BMS948 as RARβ-selective full agonist and provide rational guidelines for the development of selective antagonists.

## Results

Despite a number of gene ablation [3] and gain-of-function studies, the mechanisms underlying the specific action of RARβ have remained poorly defined. These approaches can in no way replace receptor pharmacology. Hence development of more and effective RARβ-selective retinoids is important to further dissect the function of this protein in various systems. For this purpose, novel retinoids inspired by well-characterized retinoids were prepared (Fig 1). Subsequently, and because RARs are transcription factors, the biological activity of these new synthetic retinoids was characterized by the use of transactivation reporter assays with cultured cells. Ultimately, the transcriptional activity of these compounds led us to explore the structural basis of their behavior as RAR agonist and antagonist by defining the 3D structure of the RARβ LBD complexed with these retinoids. Overall, our approach allowed refining the structure-activity relationships in both retinoid agonists and antagonists.

### BMS948 is a full RARβ-selective agonist

**Fig 2:**
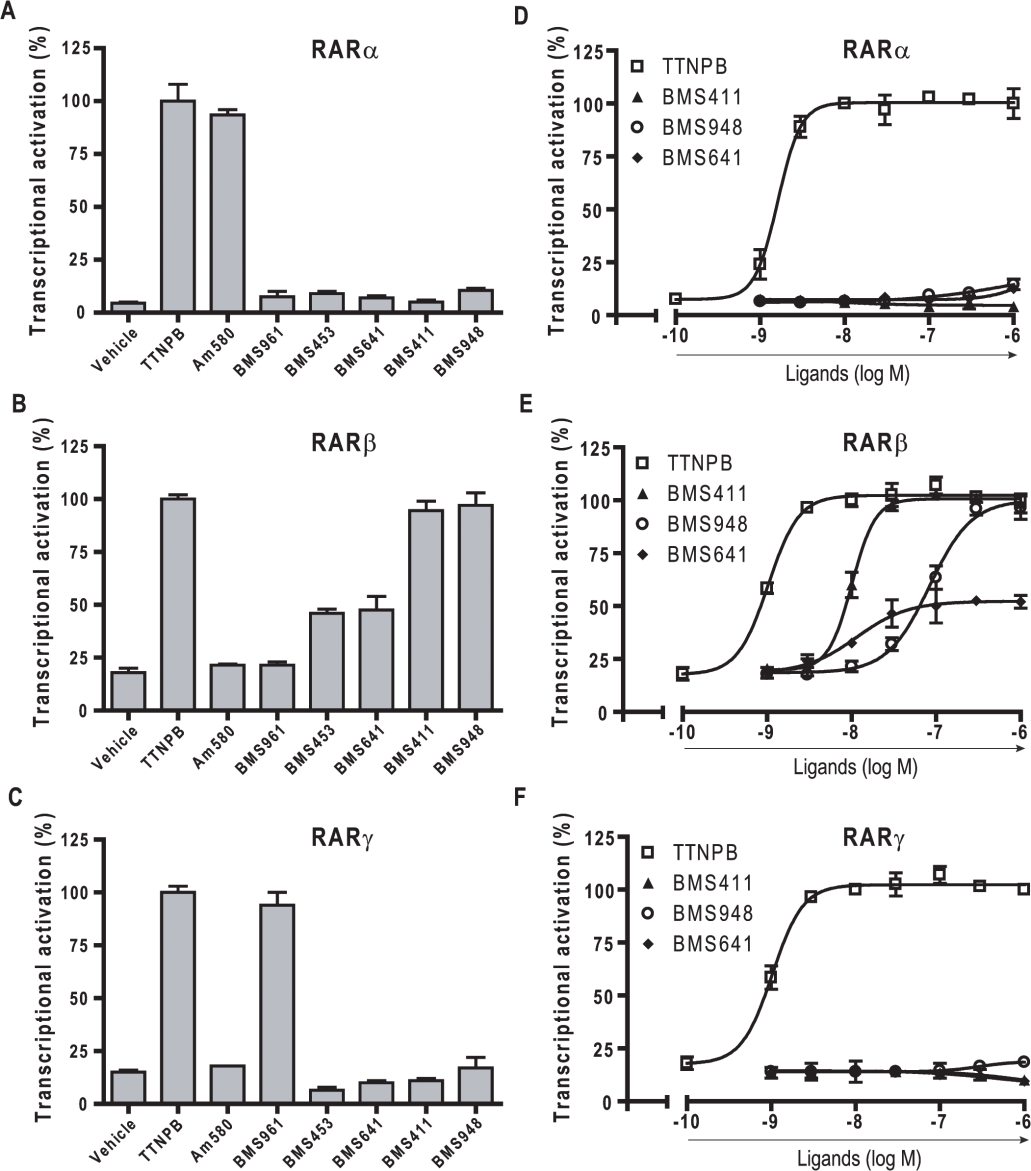
BMS948 acts as a full RARβ agonist. (A to C) HeLa cells were transiently cotransfected with the reporter (RARE)_3x_-tk-Luc and RARα (A), RARβ (B) or RARγ (C), as indicated, to assess the RAR agonist potential of synthetic RAR ligands. Cells were incubated with ligands at selective concentrations (Am580 10^–9^ M, BMS961 10^–7^ M, BMS453 10^–7^ M, BMS641 10^–7^ M, BMS411 10^–7^ M, BMS948 3.10^–7^ M). 100% corresponds to reporter gene transcription induced in the presence of the full pan-RAR agonist TTNPB at 10^–8^ M. (E to G) Dose-response curves to assess the binding affinity of synthetic RAR ligands towards RARα (E), RARβ (F), or RARγ (G). Cells were incubated with increasing concentrations of TTNPB (open squares), BMS948 (open circles), BMS411 (closed triangles), or BMS641 (closed diamonds), in a range of 10^–10^ to 10^–6^ M. All error bars are expressed as s.e.m.

**Fig 3:**
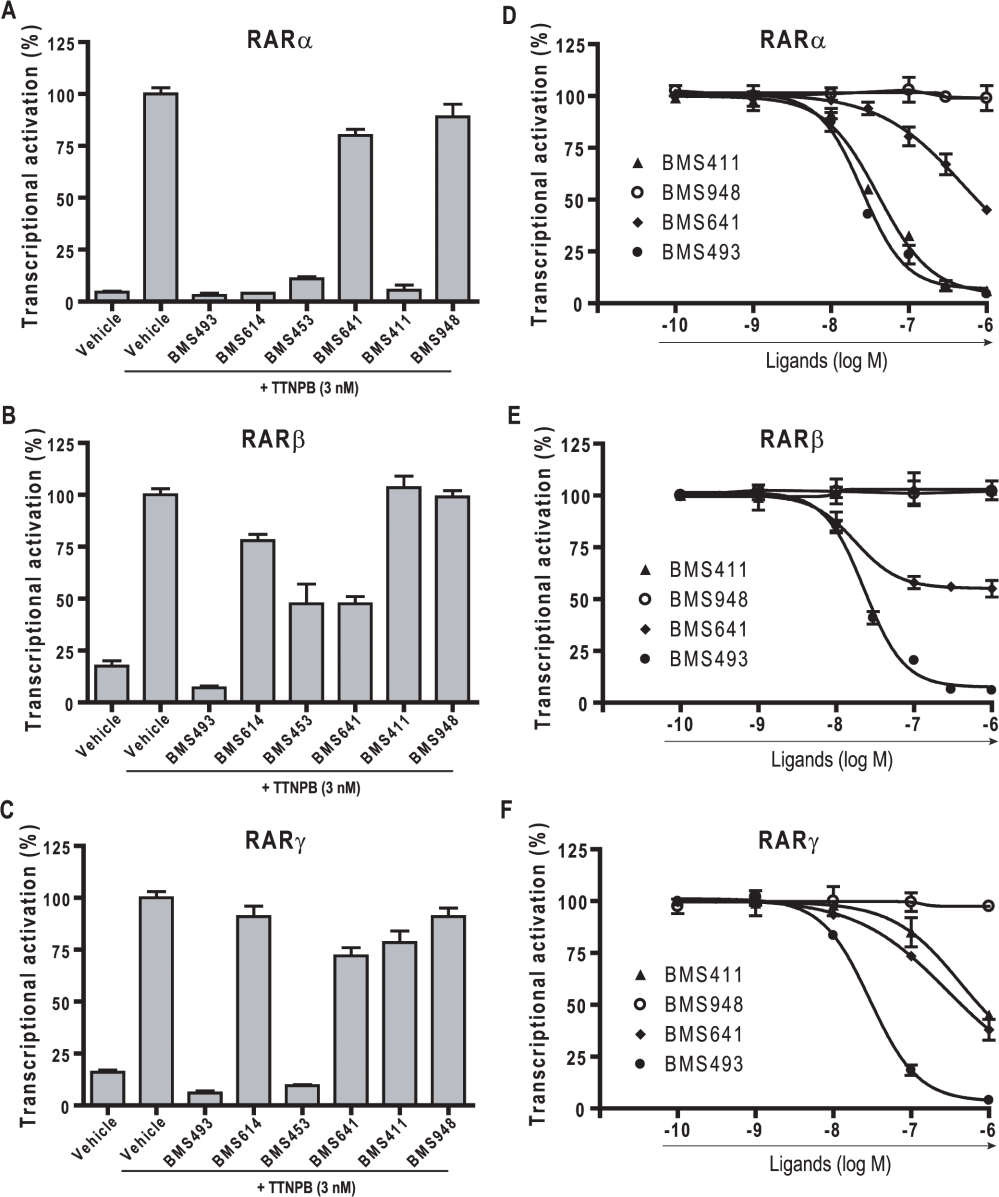
BMS948 is an RARβ-selective ligands. (A to C) Transient transactivation assays as in Fig 2 to assess the antagonist potential of synthetic RAR ligands (RARα (A), RARβ (B) or RARγ (C)). The reporter was activated with 3nM TTNPB (100%) alone and plus the synthetic retinoids added at 1 μM. (E to G) Dose-response curves (RARα (E), RARβ (F), or RARγ (G)) to assess the binding affinity of BMS493 (closed circles), BMS948 (open circles), BMS411 closed triangles), or BMS641 (closed diamonds) relative to TTNPB at 3 nM.

To evaluate the activity of a panel of synthetic retinoids (Fig 1) on RAR-mediated transactivation, we used HeLa cells transiently transfected with human RARα1,-β2, or—γ2 and a (RARE)_3x_-tk-luciferase reporter gene. Well characterized full agonist and antagonist retinoids were also used as reference compounds (S2 Fig). The transcriptional activity obtained with the various compounds was compared with that observed with 10 nM of the pan-RAR agonist TTNPB (100%), a chemically stable compound activating all RAR subtypes as efficiently as atRA (S3 Fig). In these conditions, as expected, the RARα-selective agonist Am580 at 3 nM and the RARγ-selective agonist BMS961 at 100 nM exhibited full agonistic activity for RARα and RARγ, respectively (Figs 2A–3C). Importantly, the novel compound BMS948 acted as a full RARβ-agonist at 1 μM, whereas a very weak activity was seen with RARα and RARγ (Fig 2A–2C). In comparison with previously characterized compounds, BMS948 displayed an activity profile similar to that of BMS411 [30] which also acted as an RARβ-selective full agonist whereas BMS641 [10] and BMS453 [30] acted as RARβ-selective partial agonists (Fig 2A–2C). To further characterize the activation profile of BMS compounds, transfected cells were exposed to a range of ligand concentrations to establish dose-response curves. As displayed in Fig 2D–2F, BMS411, BMS641 and BMS948 activated RARβ but very weakly RARα or RARγ, and only at the highest ligand concentration used (1 μM). Furthermore, the partial activity of BMS641 toward RARβ was confirmed as the dose-response curve plateaued at about 50% of the TTNPB-induced activity (Fig 2E). BMS948 produced a concentration-dependent increase in transactivation for RARβ with an EC50 that was about 0.1 μM and one order of magnitude higher than those determined for BMS411 and BMS641 (EC50 ≈ 10 nM) and two order of magnitude higher than of TTNPB (EC50 ≈ 1 nM). The EC50 value for a given compound has been shown to reflect its binding affinity for the target receptor [10, 30]. For instance, BMS641 which binds to RARα, RARβ and RARγ with dissociation constants (Kds) of 225 nM, 2.5 nM and 223 nM, respectively, was previously shown to activate RARβ at a 100-times lower concentration than the two other RAR subtypes [10]. We thus concluded that BMS948 must display a somewhat lower affinity for RARβ than both BMS411 and BMS641.

Having shown that BMS948 was almost ineffective in inducing transactivation through both RARα and RARγ, we tested whether this compound displayed RAR antagonistic activities. To this end, transient transactivation assays were conducted in order to assess the ability of the synthetic retinoids to affect TTNPB-induced RAR activity. In these experiments TTNPB at 3 nM was challenged with increasing amounts of the ligands and the powerful pan-RAR antagonist BMS493 at 1 μM was used as positive control for transactivation inhibition (Fig 3A–3C). As previously reported [32], BMS493 abolished the TTNPB-induced transactivation for all three RAR subtypes whereas the RARα-specific antagonist BMS614 [8, 20] counteracted the TTNPB-induced transactivation on RARα only. In the same vein, BMS453 [10, 30] antagonized almost entirely the TTNPB-induced RARα and RARγ activity and acted as a partial antagonist for RARβ, in line with its RARβ partial agonistic activity (Fig 2B). BMS411 appeared as a potent RARα antagonist with no or little antagonizing effect on RARβ and RARx, respectively. In addition, dose-response curves were conducted to evaluate the potency of molecules relative to TTNPB (Fig 3D and 3F). Competition curves with increasing concentrations of BMS411 yielded an IC50 of roughly 30 nM for RARα in keeping with the high affinity of this compound for this receptor subtype [30] (Fig 3D). Similar experiments confirmed the partial RARβ-selective antagonistic activity of BMS641 as the competition curve plateaued at about 50% of the TTNPB-induced activity. Importantly, BMS948 showed no (RARβ) or very weak (RARα and γ) inhibiting activity, suggesting that this compound binds to RARα and RARγ only at very high concentration. Overall, our data lead to the general conclusion that BMS948 is a full RARβ-selective agonist.

### Crystal structures of RARβ LBD in complex with BMS411, BMS641 and BMS948

**Fig 4:**
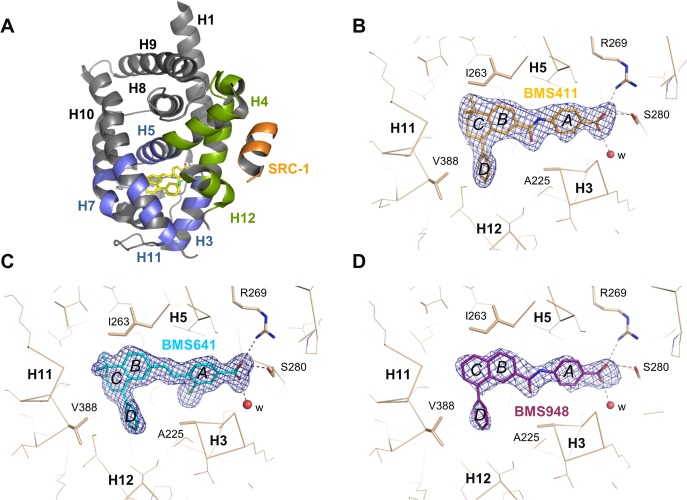
Crystallographic analysis. (A) The whole structure of the RARβ LBD in complex with BMS641 and SRC-1 coactivator peptide (in orange). The AF-2 surface formed by helices H3, H4 and H12 is highlighted in green. The lower part of the LBD, in blue, encloses the ligand-binding pocket (LBP). (B-D) The interaction networks of BMS compounds with LBD residues of RARβ are also displayed. Oxygen, nitrogen and chlorine atoms are colored in red, blue, and green, respectively. Hydrogen bonds are indicated by black dashed lines and water molecules by red spheres. The electron density represents a *F*_*o*_ *-F*_*c*_ simulated annealing omit map contoured at 3σ.

To gain structural insights into the specific binding and activity profiles of the newly characterized synthetic ligands, we solved the crystal structures of RARβ LBD in complex with BMS411, BMS641 and BMS948. To further stabilize the RARβ LBD in its active form and in turn facilitate crystallization, a peptide containing the second interaction motif of the coactivator SRC-1 was also added during the crystallization trials. Structure determination and refinement statistics are summarized in S1 Table. The structures solved at 1.9 Å (BMS641), 2.3 Å (BMS411), and 2.6 Å (BMS948) resolution display the canonical active conformation, with helix H12 capping the ligand-binding pocket (LBP) and the SRC-1 peptide bound to the so-called “AF-2 surface” formed by helices H3, H4 and H12 (as shown for the RARβ LBD–BMS641 complex in Fig 4A), thereby reflecting the agonistic character of the ligands. The compounds could be precisely placed in their respective electron density revealing a conserved binding mode. As shown in Fig 4B–4D, the ligands occupy almost the same volume in the LBP and impose similar side chain conformations. They are stabilized in the RARβ LBP through extensive van der Waals contacts and a network of ionic and hydrogen bonds between the carboxylate moiety of the retinoids, Arg_269_ in H5, Ser_280_ in the β-turn and water molecules.

### Structural features contributing to RARβ-selectivity

**Fig 5:**
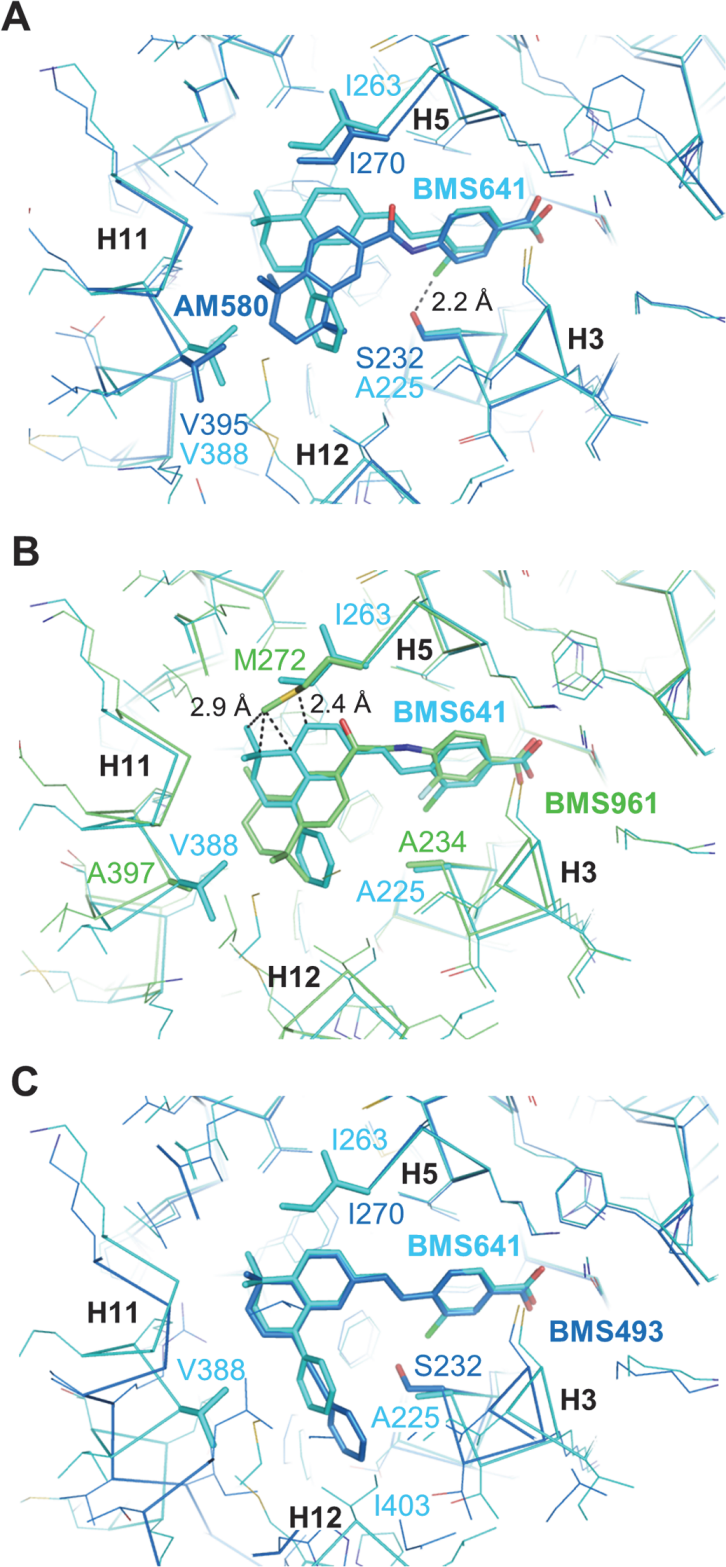
Structural features contributing to the RARβ-selectivity of BMS641. Structure superposition of BMS641-bound RARβ (cyan) with (A) Am580-bound RARα (blue), (B) BMS961-bound RARγ (green), or (C) BMS493-bound RARα (blue).

The aforementioned functional analysis identified two RARβ-selective ligands, BMS641 and BMS948. Interestingly, these retinoids display divergent linkers and chemical groups thus suggesting different recognition mechanisms. BMS641 that is closely related to both BMS453 with a chlorine atom at position C3 and UVI2007 with a phenyl group at position C8” (Fig 1), exhibited a marked RARβ-selectivity and acted as RARα/γ antagonist at high concentration only (Fig 2D–2F). This is in contrast with BMS453 and UVI2007 which acted as potent RARα/γ antagonist and RARβ partial agonist or RARβ/γ full agonist, respectively [10]. Thus we concluded that, separately, the phenyl group at position C8” or the chlorine atom at position C3 do not allow discrimination between RAR subtypes and we suggested that the selectivity toward RARβ displayed by BMS641 results from the combination of these two chemical groups. Superposition of the BMS641-bound RARβ structure onto that of RARα bound to the RARα-selective ligand Am580 revealed that the replacement of RARβ Ala_225_ by Ser_232_ in H3 of RARα generates steric hindrance that strongly reduces the binding of 3-chloro substituted compounds (Fig 5A). In the same line, comparison with the structure of RARγ bound to the RARγ-selective ligand BMS961 showed that the position of BMS641 in the LBP is incompatible with the presence of Met_272_ in RARγ (Fig 5B). Indeed, BMS641 lies in a portion of the LBP that is not occupied by the ligand in the RARα-Am580 and RARγ-BMS961 complexes. This shift in the position of BMS641 relies on the presence of the bulky C8”-phenyl ring which pushes the ligand toward RARβ Ile_263_. Of note, comparison of the BMS641-bound RARβ and BMS493-bound RARα [12] structures reveals that the position of the two stilbene-based retinoids adopt exactly the same position in RARα and RARβ LBPs (Fig 5C). Overall these observations provide a structural basis for the RARβ selectivity of BMS641 and reveal that the presence of a 3-chloro and an 8”-phenyl prevent BMS641 from binding to RARα and RARγ while maintaining a partial agonistic activity on RARβ.

**Fig 6:**
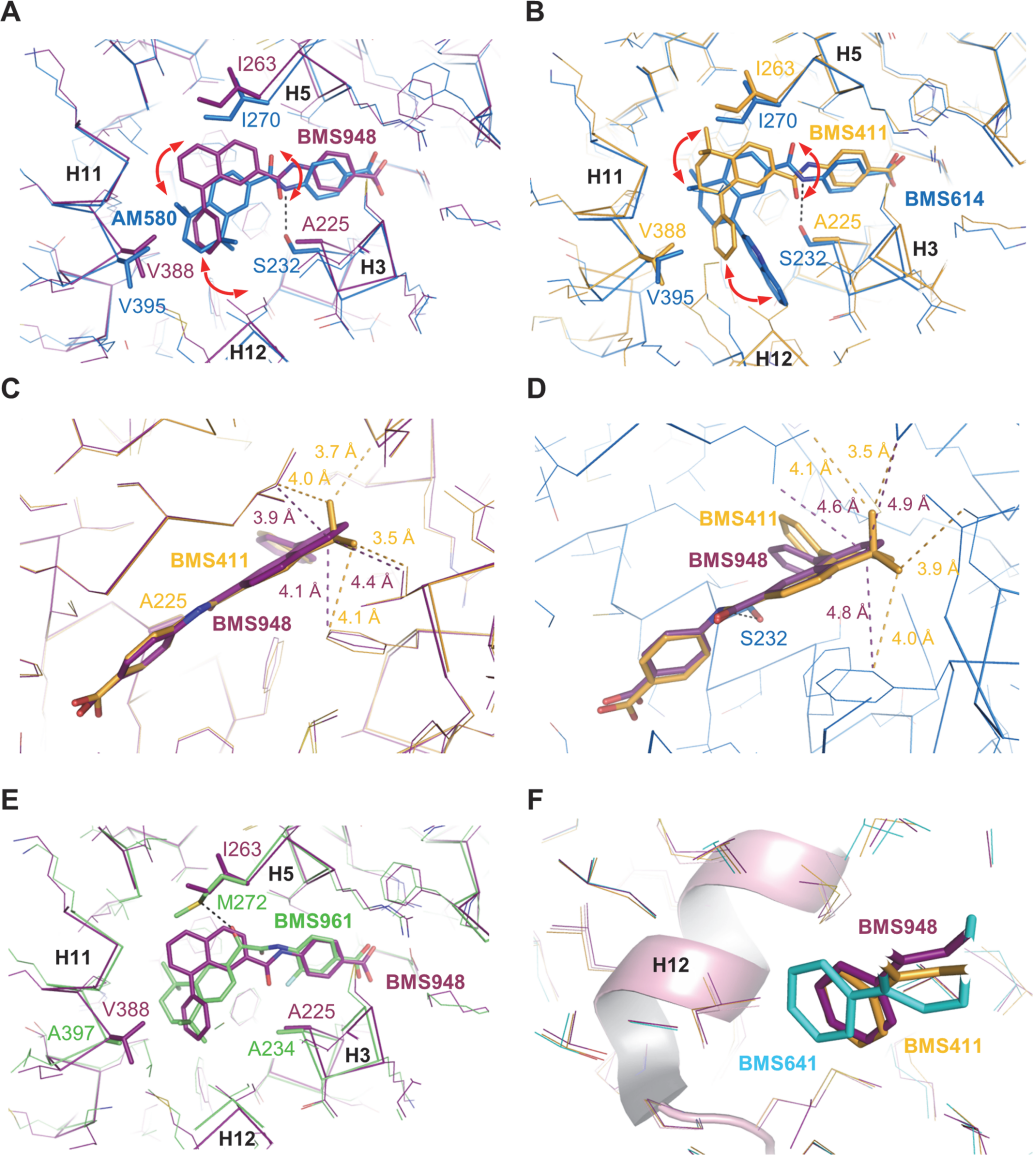
Structural features contributing to the RARβ-selectivity and full agonistic activity of BMS948. (A) Structure superposition of BMS948-bound RARβ (violet) with Am580-bound RARα (blue). (B) Structure superposition of BMS411-bound RARβ (yellow) with BMS614-bound RARα (blue). (C) Structure superposition of BMS411-bound RARβ (yellow) with BMS948-bound RARβ (violet). (D) Superposition of the docking models of BMS411 (yellow) and BMS948 (violet) in RARα. (E) Structure superposition of BMS948-bound RARβ (violet) with BMS961-bound RARγ (green). (F) Superposition of all BMS-bound RARβ structures highlighting the differential positioning of the phenyl extensions relative to helix H12. Superposition of the structures was done using the SSM function in Coot[33].

In contrast with BMS641, BMS948 harbors an amide linker (Fig 1). This is a rather unexpected observation since previous studies have revealed that retinoids with such a connector display RARα-selectivity [20, 21]. Indeed, the structures of RARα-LBD complexed with the RARα-selective agonist Am580 or the RARα-selective antagonist BMS614 (a quinolyl derivative of Am580) revealed that the amide linker of both ligands adopts a particular constrained conformation by establishing a hydrogen bond with the RARα-specific Ser_232_ through its amino moiety (Fig 6A and 6B), the carbonyl group pointing toward Ile_270_. Strikingly, the 3D structures of RARβ LBD in complex with BMS411 or BMS948 showed an alternative conformation of the amide function. In contrast to RARα, and because of the presence of an alanine residue in RARβ H3 instead of Ser_232_ in RARα, the carbonyl moiety of the amide linker of BMS948 (Fig 6A) and BMS411 (Fig 6B) points toward RARβ A_225_. This flipping by 180° of the amide bond induces a change in the position of the rings B and C of BMS948 and BMS411 as compared to that of Am580 and BMS614 in RARα. Interestingly, the only difference between BMS411, which retains a significant interaction capacity with RARα, and the RARβ-selective BMS948 resides in the replacement of a dimethyl-cyclohexenyl ring by a planar phenyl group (Fig 1). To better understand the drastic loss of affinity of BMS948 for RARα by comparison with BMS411, we used the BMS614-bound RARα structure to dock BMS948 and BMS411 in RARα and compared their LBP environment to that observed in the crystal structures of BMS948 and BMS411 bound to RARβ. Whereas the two methyl groups of BMS411 are involved in strong van der Waals interactions with LBP residues in both receptor subtypes (Fig 6C and 6D), the lack of such chemical groups in BMS948 results respectively in a modest and strong loss of van der Waals contacts between this ligand and LBP residues in RARβ and RARα. Indeed, in the absence of the two methyl groups, the carbon atom at position 5 of the BMS948 naphthalene ring remains involved in several contacts with RARβ LBP residues (Fig 6C) whereas no interaction distance below 4.6 Å is observed in RARα (Fig 6D). Thus the divergent binding modes of BMS948 in the two receptor subtypes bring the naphthalene ring into a LBP environment that is more sensitive to the loss of the methyl groups in RARα than in RARβ, in full agreement with the much higher affinity of BMS948 for RARβ. Finally, because RARγ contains an alanine residue in H3 (Ala_234_), we hypothesized that BMS948 adopts a position in this receptor subtype similar to that observed in RARβ. A superposition of the BMS948-bound RARβ LBD structure with that of RARγ bound to the selective ligand BMS961 shows that, as in the case of BMS641, the position of BMS948 is incompatible with the presence of Met_272_ in RARγ (Fig 6E). A mutational analysis in which LBPs of both RARβ and RARγ were interconverted confirmed that Met_272_ is a most important discriminatory residue (S4 Fig). Together the above considerations provide a structural rationale accounting for the RARα antagonist/RARβ agonist activities of BMS411.

### Structural basis for the specific activity of BMS ligands in RAR subtypes

Having determined the structural features accounting for the RAR subtype-selective interaction of BMS compounds, we next considered how the binding modes of BMS641, BMS411 and BMS948 may account for the divergent activity profiles of these ligands. Fig 6A and 6B highlights the prominent role of the amide linker in the differential positioning of ligands in RARα and RARβ. Given the structural analogy between BMS411 and BMS614 (Fig 1), one can reasonably make the hypothesis that both ligands adopt a similar conformation in RARα LBP. Thus, as compared to the RARβ situation, the flipping by 180° of the amide bond of BMS411 in RARα is very likely to induce a complete reorientation of the rings B and C, and bring the phenyl extension D in a position similar to that observed for the quinolyl group of BMS614 (Fig 6B), thereby interfering unfavorably with the stable positioning of H12 in the active conformation. This structural model is in full agreement with the dual RARα antagonist/RARβ agonist activities of BMS411. Overall, this structural analysis shows that the mode of binding of BMS411 to RARβ allows accommodation of the phenyl ring of the ligand within the confined environment of the active conformation. In contrast, in RARα the conformational change of BMS411 induces the reorientation of the bulky extension which projects toward H12 and prevents the active conformation of RARα from being stably formed.

We subsequently looked for a rational explanation for the differential activation of RARβ by the BMS compounds. Indeed, our transcriptional assays demonstrated that BMS641 acted as RARβ partial agonist whereas both BMS948 and BMS411 displayed full RARβ agonistic activity (Fig 2B and 2E). The superposition of all three structures reveals that, although all BMS compounds are able to lock RARβ in the active conformation which is consistent with their agonistic activity, the phenyl extension of BMS641 protrudes slightly more from the LBP toward helix H12 than that of BMS948 and BMS411 (Fig 6F). A likely consequence is that the RARβ-coactivator interaction may be less optimal in the presence of BMS641. Indeed BMS641 induced a reduced recruitment of the coactivator TIF2, accounting for the weakest agonistic activity of this compound (S5 Fig).

**Fig 7:**
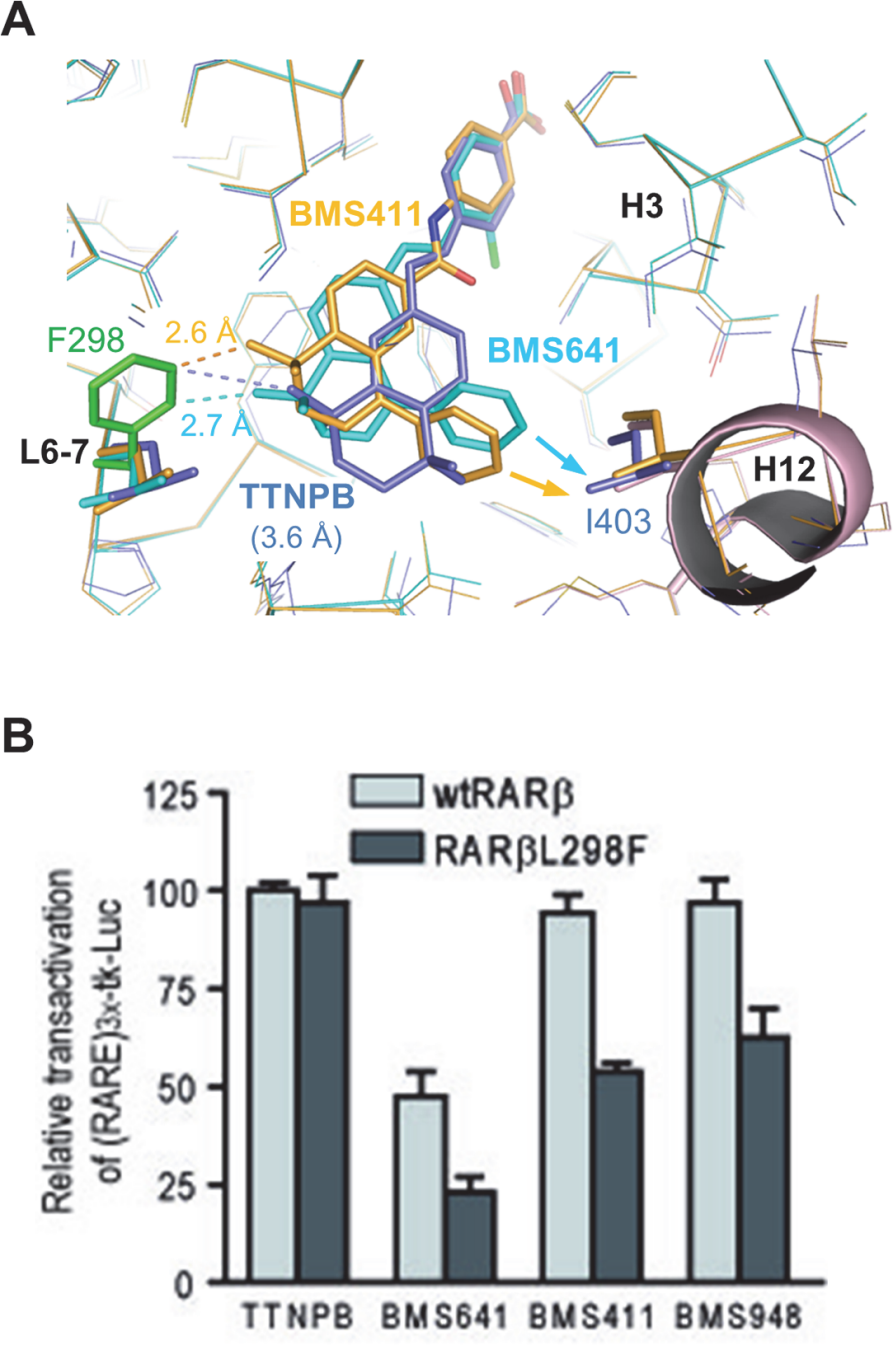
Structural model for full- and partial activity of BMS compounds. (A) Comparison of the steric clashes generated by the L_298_F mutation (green) in RARβ complexed with TTNPB (blue), BMS411 (yellow) and BMS641 (cyan). (B) Transient transactivation assays as in Fig 2 to assess the transcriptional activity of wild-type hRARβ and mutant hRARβL_298_F in the presence of the indicated retinoids or vehicle (ethanol). The reporter was activated with 10 nM TTNPB (100% with wild-type hRARβ) and the synthetic retinoids were added at 0.1 μM.

To validate experimentally our model of partial agonist action according to which the degree of agonistic/antagonistic activity varies as a function of the repulsive forces exerted by the ligand onto helix H12, we introduced a point mutation in the back of the RARβ LBP where Leu_298_ (loop between H6 and H7) was replaced by a bulkier phenylalanine residue (Fig 7A). Transient transfection assays revealed that the full agonistic activity of TTNPB was not affected by the mutation (Fig 7B). In contrast, this single mutation was detrimental for all compounds harboring a phenyl substituent at the C8” position. Whereas BMS411 and BMS948 acted as full agonists of wtRARβ, they induced only a 50–60% activity on RARβL_298_F as compared to TTNPB. Similarly, the partial agonistic activity of BMS641 was decreased from 50% (wtRARβ) to 20% (RARβL_298_F). Note that dose-response curves revealed unchanged EC50s as compared to those obtained with wtRARβ, suggesting that this reduction of the transcriptional activity is not due to a decrease of ligand affinities for the mutant (S6 Fig). Modeling of the RARβ mutant reveals that Phe_298_ generates steric clashes with rings C/D of C8” substituted compounds which is likely to provoke a displacement of the ligands toward H12 and a destabilization of the active conformation, and as a result a reduction of the binding affinity of CoA (Fig 7A). Comparison with the structure of RARβ in complex with TTNPB [34] reveals that the agonist which does not contain a bulky extension adopts a position in the LBP that allows accommodation of the phenylalanine residue without significant positional adaptation. These data substantiate the combined roles of the linker region and of the C8” substituents in the mixed agonistic/antagonistic activity of BMS compounds and provides guidelines for the design of RARβ-selective antagonists.

## Discussion

To summarize, new retinoids have been evaluated as RARβ modulators. Our results clearly showed that the dual modulatory activity of BMS411, with both full activation of RARβ and strong antagonism of RARα, is due to the presence of an amide function into its linker. This demonstrates the crucial role of the nature of the connector in both binding ability and transcriptional potential of the retinoids. In addition, BMS641, which contains a *trans*-olefin as a linker, acted as RARβ-selective partial agonist, presumably by provoking more disturbance of the H12 folding than full agonists. Finally, and strikingly, our study revealed the selectivity and the full agonistic activity of BMS948 for RARβ as well as the structural basis of these features. BMS948 is then the first RARβ-selective full agonist identified. Of note, the BMS948 selectivity is surprising as its structure contains the identical amide function as a connector than that of known RARα-selective ligand such as Am580. The unexpected structural basis of BMS948 selectivity is based on the lack of hydrophobic gem-dimethyl group located at C5” position found in both RARα ligands BMS411 (antagonist) and Am580 (agonist) which appears to be the determinant for the dramatic low affinity of BMS948 towards RARα and, as a consequence, for its RARβ-selectivity. To assess whether this absence of the two methyl groups suffices to impair the binding of a retinoid to RARα or whether the combination of this absence with the presence of a linker harboring an amide function is required, one should generate stilbene-based retinoids, such as a derivative series of BMS453, lacking the gem-dimethyl group. On the other hand, and despite a great need, RARβ antagonists have not been reported yet. Nevertheless, a diazepine fused to aryl rings, referred to as LE135 [35], has been reported as an antagonist showing some selectivity for RARβ. But, the affinity difference between RARβ and the two other RAR subtypes makes that this compound may be defined as a retinoid that only shows preference for RARβ. In addition, this compound displayed some RARγ partial agonistic activity ([35] and personal data not shown). Consequently, such a ligand is inadequate to properly investigate the function of this protein, mainly in the context of *in vivo* analyses. Hence the present challenge is to integrate our above molecular and structural information to provide potent and selective RARβ antagonists. A possible strategy to achieve this goal is to start from the RARβ-selective ligands identified in this work, namely BMS641 and BMS948. Because the reported crystal structures of RARs [8, 10, 12] have showed that substitution at the naphthalene C8 position endow in general ligands with RAR antagonist activities, a further extension of the substituent at this position of both BMS641 and BMS948 could impact on H12 positioning and thus afford ligands with antagonist properties. In this respect, an entire series of C3-halogenated derivatives and bulkier analogues at C8” position of the parent stilbene-based BMS641 has been generated and characterized in term of their RAR transcriptional potential [36]. Whereas most of these ligands acted as potent RARβ antagonists, the RARβ-selectivity observed for BMS641 is lost in the presence of bulkier substituents. Then, one potential caveat is that an increase in bulk may change the rules defining the binding selectivity. More recently, inspired in the parent BMS948, a C5,C8-diphenylnaphtalene-2-yl linked to a benzoic acid via an amide connector has been reported [37]. Unfortunately, this compound did not exert an RAR antagonistic activity, but exhibited a RARβ agonistic activity at very high concentration (10 μM).

Importantly, we have observed that replacement of the phenyl group at position C8” of the RARβ agonist BMS411 by a *p*-tolyl group, yielding BMS532 (Fig 1), converted the ligand into a potent antagonist for RARβ, while BMS532 retained a high affinity for RAR03B1 (data not shown). Together with the fact that both BMS411 and BMS948 adopt the same conformation in the RARβ LBP and that the absence of the gem-dimethyl group at C5” position is a crucial point to lose affinity towards RARα, these considerations led us to predict that increasing the bulkiness of the hydrophobic group of BMS948-like molecules, notably by introducing a *p*-tolyl group instead of the phenyl group, would generate retinoids that sterically interfere with the agonist positioning of H12 and thus turn into RARβ–selective antagonists.

In conclusion, the novel RARβ-selective full agonist presented here should represent a useful tool for both developing selective antagonists for RARβ and pharmacologically addressing the specific RARβ function *in vitro* and in animal models, and possibly, therapeutic exploitation. In addition, our overall results demonstrate that subtle changes in the chemical structure of the ligand or in the residues lining the LBPs translate in very selective effects on the transcriptional potential of the receptor.

## Materials and Methods

### Ligands and plasmids

BMS compounds (BMS493 [32], BMS614 [20], BMS411 [20], BMS948, BMS961 [9]) were provided by Bristol-Myers Squibb. TTNPB was purchased from Sigma. Am580 was kindly provided by Reinhold Tacke (University of Würzburg). pSG5-based RAR expression vectors were described previously (19). All ligands are in ethanol solutions. (RARE)_3x_-tk-luc was a kindly gift of Patrick Balaguer (INSERM, Montpellier). hRARβL_298_F was generated into pSG5-hRARβ by PCR-assisted site-directed mutagenesis with Deep Vent DNA polymerase (New England Biolabs). The construct was verified by DNA sequencing.

### Cell culture and transient transfections

HeLa cells were cultured in DMEM with Glutamax and 10% (v/v) FCS and transfected using JetPei transfectant (Ozyme). After 24 h, the medium was changed to a medium containing the indicated ligands or vehicle. Cells were lysed and assayed for reporter expression 48 h after transfection. The luciferase assay system was used according to the manufacturer’s instruction (Promega). In each case results were normalized to coexpressed β–galactosidase. Each transfection was carried out in duplicate and repeated each three to six times.

### Protein production, purification and crystallization

The human wild-type RARβ LBD (amino acids 169–414) was cloned into the pET-15b vector and expressed in *Escherichia coli* BL21(DE3) cells. Cells were grown at 37°C in LB medium supplemented with 50 mg.mL^-1^ ampicillin until OD_600_ reached about 0.6. Expression of T7 polymerase was induced by addition of isopropyl-β-D-thiogalactoside (IPTG) to a final concentration of 0.5 mM. After an additional incubation for 8 h at 20°C, cell cultures were harvested by centrifugation at 8,000 g for 20 min. Cell pellets from 2 L of culture were resuspended in 50 mL buffer-A (20 mM Tris-HCl pH 7.5, 500 mM NaCl, 1 mM DTT) supplemented with a protease inhibitor cocktail (cOmplete, Mini, EDTA-free; Roche Applied Science). The suspension was lysed by sonication and centrifuged at 4°C for 45 min. The supernatant was loaded onto a nickel affinity column (HisTrap 5 mL; GE Healthcare) pre-equilibrated with buffer-A. The protein was eluted with buffer-B (20 mM Tris-HCl pH 7.5, 500 mM NaCl, 1 mM DTT, 500 mM Imidazole). The fractions containing hRARβ LBD were pooled and further purified by size exclusion chromatography (Superdex 75 HR 26/60; GE Healthcare). Prior to crystallization the purified hRARβ LBD was further complexed with 2 equimolar of ligand (BMS948, BMS641, and BMS411 provided by Bristol-Myers Squibb) and 3 equimolar of SRC-1 co-activator peptide (the RHKILHRLLQEGS peptide corresponding to the NR box 2-binding motif was purchased from EZbiolab). The complexes were concentrated to 10 mg.ml^-1^ in the gel filtration buffer (20 mM Tris pH 7.5, 150 mM NaCl, 5 mM DTT and 1 mM EDTA). The various RARβ-ligand-SRC-1 complex crystals were obtained in 200 mM Trisodium citrate pH 5.5 and 25% PEG 4000 using the hanging drop crystallization method.

### Data collection and structure determination

Native data were collected from crystals cryoprotected with 30% glycerol on the BM30A and ID14-2 beamlines at the European Synchrotron Radiation Facility, Grenoble, France. Data were processed and scaled using XDS and XSCALE [38]. Crystals belong to space group *P*2_1_2_1_2_1_ for all complexes. Structures were solved by molecular replacement method using PHENIX (phenix.automr) [39], and refinement and rebuilding were performed with COOT [33], PHENIX (phenix.refine) [39] and REFMAC [40] from the CCP4 suite [41]. Data collection and refinement statistics are summarized in Supporting Information. Figures were prepared with PyMOL (http://pymol.org/).

### Accession codes

Protein Data Bank: Atomic coordinates and structure factors for RARβ-BMS948-SRC-1 NR2, RARβ-BMS411-SRC-1 NR2, and RARβ-BMS641-SRC-1 NR2 complexes have been deposited under accession codes 4JYH, 4JYG, and 4JYI, respectively.

## Supporting Information

S1 Fig Determination of the BMS948 purity by HPLC/LCMS analysis. The purity of BMS948 was determined by LCMS analysis, which was performed on a system consisting of an electrospray source on a Waters Micromasss ZQ mass spectrometer, a Waters 2996 diode array detector, a Waters alliance 2695 HPLC system with autosampler, and a Macherey-Nagel Nucleoshell RP18 plus HPLC column (5 μm, 4 mm [1] 100 mm). The HPLC method incorporated UV detection on a range of 214–400 nm, a column temperature of 40°C, a flow rate of 1.0 mL/min, a 50 μL injection volume, and a binary solvent system of 0.1% formic acid in water (solvent A) and 0.1% formic acid in CH3CN (solvent B). The following gradient (10 min) was used: 0–1 min, 5% B; 1–7 min, 5–100% B; 7–10 min, 100% B. (A) BMS948 was isolated to >98% purity (98.49%). (B) MS spectrum confirmed the presence of BMS948.
(EPS)

S2 Fig Dose-response curves for reference retinoids. (A to C) Transient transactivation assays as in Fig 2 (RARα (A), RARβ (B), or RARγ (C)). Cells were incubated with increasing concentrations of TTNPB (open squares), Am580 (closed triangles), BMS961 (closed circles), or BMS453 (open circles), in a range of 10^–10^ to 10^–6^ M. All error bars are expressed as s.e.m. (E to G) Transient transactivation assays as in Fig 3 to assess the antagonist potential of synthetic retinoids (RARα (E), RARβ (F) or RARγ (G)). The reporter was activated with 3nM TTNPB (100%) alone and plus the synthetic retinoids (BMS614 (closed triangles), BMS453 (open circles), BMS493 (closed circles) in a range of 10^–10^ to 10^–6^ M.
(EPS)

S3 Fig TTNPB and *all trans* retinoic acid exhibit a similar efficacy. HeLa cells were transiently cotransfected with the reporter (RARE)_3x_-tk-Luc and RARα (black bars), RARβ (light grey) or RARγ (dark grey), as indicated, to assess the RAR agonist potential of *all trans* retinoic acid (atRA) and TTNPB at 10 nM.
(EPS)

S4 Fig The methionine 272 of RARγ LBP is a most important discriminatory element for the RARβ-specific response to BMS948. A sequence analysis of RARβ- and RARγ-LBPs showed that a leucine residue (βIle_263_) in RARβ is replaced by a methionine residue (γMet_272_) in RARγ. To investigate the role of these residues in the RARβ–specific response to BMS948, we compared the agonistic and antagonistic potentials of this compound for RARβ and RARγ, as well as chimeric RAR mutants in which LBPs were interconverted (denoted RARβ→γ and RARγ→β). Dose-response curves were established from transient transfection in HeLa cells of these proteins and a (RARE)_3x_-tk-luciferase reporter gene as described in Figs 2 and 3. When the LBP of RARγ was converted into that of RARβ, RARγ→β responded to BMS948 like RARβ did, that is BMS948 acted as a potent full agonist, whereas a very weak activation was seen only at high concentration for RARβ→γ and the parental RARγ. In addition, BMS948 did not reduce TTNPB-induced activity for RARβ→γ which harbors a methionine residue. Overall these results show that by changing the RARγ LBP into that of RARβ, RARγ→β acquired the ability to bind BMS948, demonstrating that the replacement of γMet_272_ with a leucine residue most likely accounts for the acquisition of BMS948-binding ability by the mutant RARγ→β. These results underscore the importance of these divergent residues for the selectivity of BMS948 toward RARβ and suggest that γMet_272_ is the most important discriminatory element, in full agreement with our structural analysis (Fig 5B). (A) HeLa cells were transiently cotransfected with the reporter (RARE)_3x_-tk-Luc and RARβ (open circles), RARγ (closed diamonds), RARγ→β in which the methionine residue 272 in RARγ LBP is replaced by an isoleucine residue (open squares), or RARβ→γ in which the isoleucine residue 263 in RARβ LBP is replaced by a methionine residue (closed triangles) to assess the agonist potential of BMS948. Cells were incubated with increasing concentrations of BMS948 in a range of 10^–9^ to 10^–6^ M. 100% corresponds to reporter gene transcription induced in the presence of the full agonist TTNPB at 10nM. (B) Transient transfection assays in HeLa cells with the reporter (RARE)_3x_-tk-Luc and RARβ→γ as in (A) to assess the antagonist potential of BMS948 and BMS493. The reporter was activated with 3 nM TTNPB (100%) alone and plus the BMS compounds at 1 μM.
(EPS)

S5 Fig Molecular basis of BMS641 partial activity. According to the current model of gene regulation by RARs, the agonistic property of a given retinoid depends on its ability to induce coregulator recruitment. RAR agonists dissociate corepressors (such as SMRT) and induce the recruitment coactivators (such as TIF2), resulting in transactivation. The transcriptional data reported in Figs 2 and 3 revealed that BMS641 acts as an RARβ agonist with reduced efficacy (50%) when compared to the full agonist TTNPB (100%). BMS641 was therefore categorized as a partial RARβ agonist. To further characterize the partial activity of BMS641, we used two-hybrid assays to monitor the interaction between RARβ and transcriptional coregulators in the presence of various retinoids. In these assays, a chimeric luciferase-based reporter gene ((17 m)_5x_-βGlob-Luc) is transfected together with two expression vectors. One expresses a fusion protein (Gal-TIF2 or Gal-SMRT) that binds through the Gal DNA binding domain (Gal) to the pentamer of the “17m” DNA recognition site in the reporter gene and contains the nuclear receptor interacting domain of the coregulator. The other vector expresses a second fusion protein composed of the VP16 acidic transcription activation domain, and the LBD of RARβ. If a ligand induces RARβ-coregulator interaction, this results in the indirect recruitment of the VP16 activation domain to the promoter of the luciferase reporter gene and to the luciferase protein synthesis which can be easily quantified by using a luminometer. Two-hybrid analyses using Gal-TIF2 indicated that the RARβ-TIF2 interaction was optimal in the presence of the full RARβ agonists TTNPB, BMS411, and BMS948, while only a partial effect was seen with BMS641 (75%). As expected, BMS493 prevented the recruitment of TIF2 by RARβ. Regarding corepressor interaction, SMRT binding was decreased in the presence of all three full RARβ agonists and strongly enhanced in the presence of BMS493, in keeping with the inverse agonistic property of this ligand. Strikingly, the RARβ-SMRT interaction was maintained in the presence of BMS641, in agreement with the partial agonistic activity of this compound. Overall these data indicated that ligands show distinct pattern of RARβ-coregulator interaction which apparently account for their transcriptional activity. Retinoids characterized as full agonists (TTNPB, BMS411, and BMS948) are able to dissociate the corepressor SMRT and to induce efficient recruitment of the coactivator TIF2, consistent with their transcriptional efficacy. BMS641 induces reduced coactivator recruitment, but cannot dissociate corepressors. Consequently, as the binding of coactivators and corepressors are mutually exclusive, the resulting transcriptional activity led by BMS641 is partial. Thereby BMS641 can be classified as a potent partial agonist on the basis of its reduced efficacy relative to full agonists that originates from its pattern of coregulator interaction. Mammalian two-hybrid assays with (17m)_5x_-βGlob-Luc and Gal-TIF2 (A, 100% corresponds to reporter gene transcription induced by 10 nM TTNPB) or Gal-SMRT (B, 100% corresponds to reporter gene transcription in the absence of ligand) as bait and VP16-RARβLBD as prey were performed in HeLa cells to assess the influence of BMS compounds at 1 μM (compared to TTNPB 10 nM) on interaction between RAR and both coregulators TIF2 and SMRT in a cellular context.
(EPS)

S6 Fig The single mutation L298F in the RARβ LBP does not affect ligand affinities relative to the parental RARβ. A point mutation was introduced in the RARβ LBP where Leu_298_ was replaced by a bulkier phenylalanine residue (Fig 7A). Transient transfection assays revealed that, whereas the efficacy of TTNPB was not affected by the mutation (Fig 7B), this single mutation was detrimental for all BMS compounds harboring a phenyl substituent at the C8” position. Dose-response curves show a concentration-dependent increase in activation for RARβL_298_F with however a reduction of the efficacy of all BMS compounds (70%, 50%, and 25% for BMS948, BMS411, and BMS453, respectively) and, in contrast, unchanged EC50s when compared to those measured with the parental RARβ (Fig 2E), thus suggesting that the reduction of the transcriptional activity for RARβL_298_F is not due to a decrease of ligand affinities for this mutant. HeLa cells were transiently cotransfected with the reporter (RARE)_3x_-tk-Luc and the RARβL298F mutant (in which the phenylalanine residue 298 is replaced by a leucine residue in the RARβ LBP) to assess the RAR agonist potential of synthetic RAR ligands. Cells were incubated with increasing concentrations of TTNPB (open squares), BMS948 (open circles), BMS411 (closed triangles), or BMS641 (closed diamonds). Gal-TIF2 [32], Gal-SMRT [6], and pSG5-based RAR [20] expression vectors were described previously.
(EPS)

S1 Table Data collection and refinement statistics. (DOCX)
