# Supplementary material for: An Unexpected Mode Of Binding Defines BMS948 as A Full Retinoic Acid Receptor β (RARβ, NR1B2) Selective Agonist
Source: PLoS One. 2015 May 1;10(5):e0123195. doi: 10.1371/journal.pone.0123195 (PMC4416907; doi:10.1371/journal.pone.0123195)
Supplement: S1 Table — (DOCX) [file pone.0123195.s007.docx]

**Table S1: Data collection and refinement statistics**

|  | **RARβ_BMS641**  Pdb code 4JYI | **RARβ_BMS411**  Pdb code 4JYG | **RARβ_BMS948**  Pdb code 4JYH |
| --- | --- | --- | --- |
| **Data collection** |  |  |  |
| Space group | *P* 2_1_2_1_2_1_ | *P* 2_1_2_1_2_1_ | *P* 2_1_2_1_2_1_ |
| Cell dimensions |  |  |  |
| a, Å | 58.52 | 58.24 | 58.30 |
| b, Å | 84.24 | 84.83 | 84.14 |
| c, Å | 109.29 | 108.42 | 108.86 |
| α, ° | 90.00 | 90.00 | 90.00 |
| β, ° | 90.00 | 90.00 | 90.00 |
| γ, ° | 90.00 | 90.00 | 90.00 |
| Resolution, Å | 48.0 – 1.9  (2.0 - 1.9)* | 48.0 – 2.3  (2.5 - 2.3)* | 47.9 – 2.6  (2.7 - 2.6)* |
| *R*_sym_, % | 4.8 (38.2) | 5.7 (28.9) | 8.5 (44.6) |
| *I*/σ*I* | 20.2 (3.3) | 19.6 (3.8) | 11.6 (3.3) |
| Completeness, % | 99.2 (96.1) | 97.1 (85.2) | 98.8 (98.5) |
| Redundancy | 4.7 (4.1) | 4.6 (3.5) | 3.7 (3.4) |
|  |  |  |  |
| **Refinement** |  |  |  |
| Resolution, Å | 39.30 – 1.90 | 39.50 – 2.30 | 42.07 – 2.60 |
| No. of reflections | 42,949 | 22,371 | 16,870 |
| *R* / *R*_free_ | 0.196 / 0.227 | 0.198 / 0.257 | 0.220 / 0.267 |
| *B*_factors_, Å^2^ |  |  |  |
| All | 32.5 | 40.8 | 44.0 |
| Protein | 31.8 | 40.8 | 44.2 |
| Ligands | 26.6 | 41.2 | 37.6 |
| Water | 40.6 | 42.9 | 41.9 |
| Rmsd |  |  |  |
| Bond lengths, Å | 0.004 | 0.005 | 0.004 |
| Bond angles, ° | 0.895 | 1.133 | 0.937 |

*The values in parentheses are for the highest-resolution shell.
